# Liver stiffness assessed by elastography for the evaluation of hepatocellular carcinoma risk in patients with fibrosis: a systematic review and meta-analysis

**DOI:** 10.1590/0100-3984.2024.0108

**Published:** 2025-06-02

**Authors:** Marielle Malucelli Mallmann, Fernando Linhares Pereira, Maria Cristina Chammas, Márcio Luís Duarte, Ana Maria Andrade, Emilton Lima Júnior, Odery Ramos Júnior

**Affiliations:** 1 Universidade Federal do Paraná (UFPR), Curitiba, PR, Brazil; 2 Instituto de Radiologia do Hospital das Clínicas da Faculdade de Medicina da Universidade de São Paulo (InRad/HC-FMUSP), São Paulo, SP, Brazil; 3 Universidade de Ribeirão Preto (Unaerp), Campus Guarujá, Guarujá, SP, Brazil; 4 Diagnósticos da América S.A. (Dasa), São Paulo, SP, Brazil

**Keywords:** Carcinoma hepatocelular, Técnicas de imagem por elasticidade, Neoplasias hepáticas/diagnóstico por imagem., Carcinoma, hepatocellular, Elasticity imaging techniques, Liver neoplasms/diagnostic imaging.

## Abstract

This study aimed to systematically review the highest-quality evidence regarding the cutoff value in kPa for the diagnostic accuracy of ultrasound-based liver elastography in comparison with reference standards, including magnetic resonance imaging (MRI), computed tomography, and liver biopsy. In addition, we assessed the presence of hepatocellular carcinoma (HCC) and its associated implications in clinical and diagnostic contexts. We conducted a search using Medical Subject Headings across PubMed, Embase, Cochrane Library, Web of Science, Scopus, and Lilacs for articles published up to June 6, 2024. Of 1,131 studies identified, 33 were eligible and 8 met the quality criteria, as evaluated with the “RTI Item Bank” and “QUADAS-2” tools, following the PICO strategy. The mean elasticity of the liver parenchyma among patients with confirmed HCC was 18.77 kPa (95% CI: 16.28-21.27), making ultrasound liver elastography useful as a predictor of the diagnosis by gold-standard methods such as MRI. Ultrasound elastography is a low-cost, accessible, and noninvasive diagnostic tool capable of estimating liver elasticity in patients with HCC. However, due to the heterogeneity of the articles included in this review, further prospective studies are needed in order to confirm and standardize a cutoff stiffness value for early HCC screening, which could improve patient outcomes, particularly in resource-limited settings.

## Abstract (pt)

Este estudo teve como objetivo revisar, sistematicamente, as melhores evidências disponíveis sobre o valor de corte em kPa para o rendimento diagnóstico da elastografia hepática por ultrassom em comparação com métodos de referência, como a ressonância magnética, a tomografia computadorizada e a biópsia hepática, além de avaliar a presença de carcinoma hepatocelular (CHC) e suas implicações no contexto clínico e diagnóstico. Uma busca utilizando os termos Medical Subject Headings foi realizada nas bases PubMed, Embase, Cochrane Library, Web of Science, Scopus e Lilacs para artigos publicados até 6 de junho de 2024. De 1.131 estudos, 33 foram considerados elegíveis e 8 atenderam aos critérios de qualidade usando as ferramentas “RTI Item Bank” e “QUADAS-2”, seguindo a estratégia PICO. A elasticidade média do parênquima hepático foi 18,77 kPa (IC 95%: 16,28-21,27) para a detecção de CHC em comparação à ressonância magnética. A elastografia hepática por ultrassom é uma ferramenta diagnóstica de baixo custo, acessível e não invasiva, capaz de estimar a elasticidade hepática em pacientes com CHC. No entanto, em razão da heterogeneidade desta revisão, mais estudos prospectivos são necessários para confirmar e padronizar um valor de corte em kPa para a triagem precoce do CHC, o que poderia melhorar os resultados dos pacientes, especialmente em ambientes com recursos limitados.

## INTRODUCTION

Hepatocellular carcinoma (HCC), a primary liver tumor, is among the fastest-growing causes of cancer-related mortality worldwide, accounting for 8.3% of all cancer deaths, according to the Global Cancer Observatory^**(^1^)**^. Liver fibrosis increases the annual risk of HCC, from less than 1% in individuals without fibrosis to 3-7% in those with cirrhosis, which is seen in 90% of individuals with HCC in Western countries^**(^2^)**^. Other major risk factors for HCC include hepatitis B, hepatitis C, advanced age, male sex, alcohol consumption, and aflatoxin exposure^**(^3^)**^, as well as obesity, metabolic dysfunction-associated steatotic liver disease (MASLD), and nonalcoholic steatohepatitis, all of which elevate the risk of cirrhosis because of chronic inflammation and fibrosis, further increasing the likelihood of HCC^**(^4^,^5^)**^. Despite the risk, fewer than a third of patients participate in regular screening for HCC^**(^5^)**^, resulting in delayed detection and a generally poor prognosis. The five-year survival rate is a mere 18%^**(^2^,^6^)**^. To improve patient outcomes, hepatology societies advise a follow-up examination every six months for individuals with cirrhosis or significant (≥ F3) fibrosis^**(^7^)**^. Diagnostic tools commonly employed for HCC include magnetic resonance imaging (MRI), computed tomography (CT), and contrast-enhanced ultrasound, although the last may not identify all lesions^**(^8^)**^. Examinations by MRI and CT are expensive and not widely available^**(^9^)**^, and some patients may encounter issues such as claustrophobia or have contraindications to contrast agents^**(^10^,^11^)**^. Although biopsy provides an accurate diagnosis, it poses risks like tumor seeding and sampling errors^**(^12^)**^.

**Figure 1:**
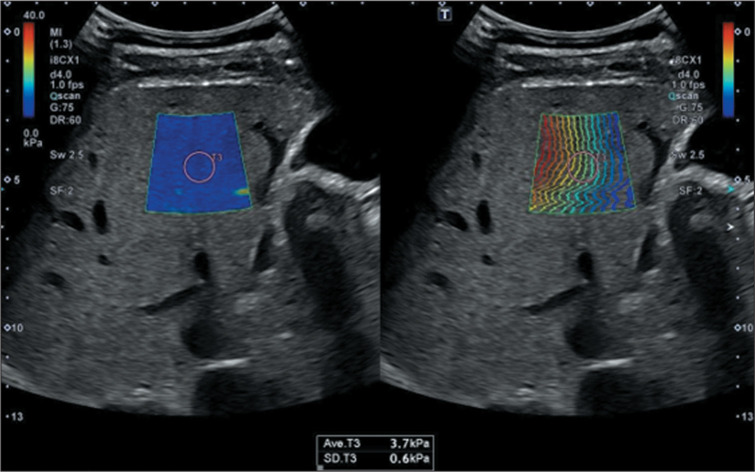
2D-SWE assessment of liver elasticity in MASLD without evidence of fibrosis.

**Figure 2:**
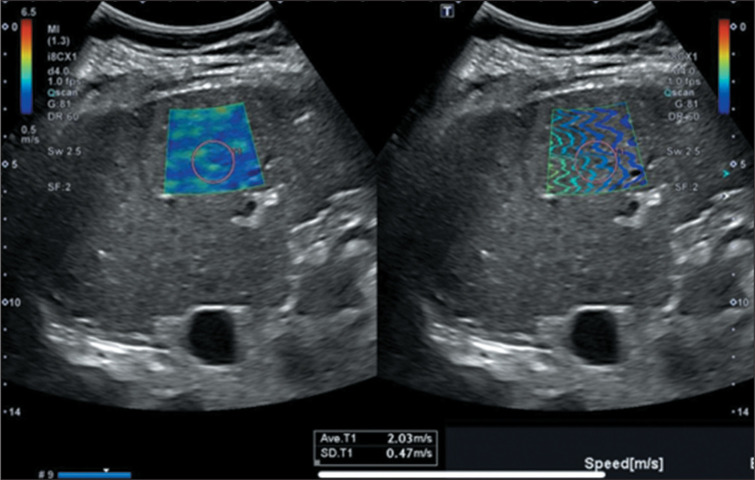
Increased propagation speeds of shear waves, representing cirrhosis.

Ultrasound elastography of the liver is an emerging, noninvasive technique for evaluating liver stiffness and tracking patients at elevated risk^**(^8^)**^. This technique can involve the use of point shear-wave elastography (p-SWE), two-dimensional shear-wave elastography (2D-SWE), or vibration-controlled transient elastography^**(^13^)**^. The stiffness of the liver, expressed in meters per second or in kPa, is directly linked to the risk of developing HCC, with each unit increase in kPa increasing the risk by 4%^**(^8^)**^. Liver elasticity can be evaluated by p-SWE or 2D-SWE. Illustrative examples are presented in Figure 1, which demonstrates liver elasticity in MASLD without evidence of fibrosis, and Figure 2, which depicts increased shear wave propagation velocities, indicative of cirrhosis.

Because of the high costs associated with MRI and CT scans^**(^14^)**^, together with the drawbacks of contrast-enhanced ultrasound and biopsy, elastography is gaining traction as a practical option for regular monitoring and early detection of HCC. It is particularly recommended for individuals with cirrhosis, to enhance treatment outcomes^**(^9^,^14^)**^.

The aim of this review was to systematically identify, analyze, and summarize the best available evidence on the cutoff value in kPa for the diagnosis of HCC by ultrasound elastography of the liver, comparing its performance with that of MRI, CT, or liver biopsy.

## METHODS

This systematic review was registered with the Center for Open Science (identifier: https://doi.org/10.17605/OSF.IO/PTZM9) and was exempt from the requirement for informed consent. The study design adhered to the guidelines outlined in the Cochrane Handbook for Systematic Reviews of Diagnostic Test Accuracy^**(^15^)**^. No funding or external support was provided for this study.

Adults ≥ 18 years of age, with either suspected or confirmed HCC, were included in this study, regardless of the severity or duration of the disease. All participants underwent elastography, with MRI, CT, or biopsy serving as the reference standard.

A comprehensive systematic literature search was conducted on June 6, 2024 across the PubMed, Embase, Cochrane Library, Web of Science, Scopus, and Lilacs databases. Additional references were identified by cross-referencing bibliographies of relevant studies and review articles. The search strategy included original publications using Medical Subject Headings terms, and characteristics of the patients included are provided in Table 1.

**Table 1:** Search strategy used in the PubMed, Embase, Cochrane Library, Web of Science, Scopus, and Lilacs databases.

Database	Search strategy
BVS	(‘liver cell carcinoma’/exp) AND (‘shear wave elastography’/exp (AND NOT ‘transient elastography’/exp OR FibroScan)) AND (‘nuclear magnetic resonance imaging’/exp OR ‘liver biopsy’/exp)
Scopus	(((hepato^*^ OR liver) AND (carcinom^*^ OR cancer OR neoplasm^*^ OR tumor^*^) OR hepatoma) AND (“Elasticity Imaging Techniques” AND NOT (transient OR fibroscan)) AND (“Magnetic Resonance Imaging” OR tomography OR (biops^*^ AND (imag^*^ OR surger^*^))) AND (elasticity OR stiffness))
PubMed	((Hepato^*^ OR liver) AND (carcinom^*^ OR cancer OR neoplasm^*^ OR tumor^*^) OR Hepatoma) AND (“Elasticity Imaging Techniques” NOT (transient OR FibroScan)) AND (“Magnetic Resonance Imaging” OR tomography OR (biops^*^ AND (imag^*^ OR surger^*^))) AND (Elasticity OR stiffness)
Embase	(‘liver cell carcinoma’/exp) AND (‘shear wave elastography’/exp (AND NOT ‘transient elastography’/exp OR FibroScan)) AND (‘nuclear magnetic resonance imaging’/exp OR ‘liver biopsy’/exp)
Web of Science	(((hepato^*^ OR liver) AND (carcinom^*^ OR cancer OR neoplasm^*^ OR tumor^*^) OR hepatoma) AND (“Elasticity Imaging Techniques” AND NOT (transient OR fibroscan)) AND (“Magnetic Resonance Imaging” OR tomography OR (biops^*^ AND (imag^*^ OR surger^*^))) AND (elasticity OR stiffness))

BVS, Biblioteca Virtual em Saúde (Brazilian Virtual Health Library).

### Selection criteria

The study adhered to the guidelines established by the Preferred Reporting Items for Systematic Reviews and Meta-Analyses^**(^16^)**^, and the research question was formulated by using the PICO framework^**(^17^)**^, as outlined below:

• Patients of interest: patients diagnosed with HCC
• Intervention to be studied: SWE examinations (p-SWE or 2D-SWE) during the study period
• Comparison of intervention: MRI, CT, or biopsy
• Outcome of interest: diagnostic effectiveness of SWE in terms of sensitivity, specificity, positive predictive value, negative predictive value, and overall accuracy in detecting HCC.

The guiding question^**(^18^-^20^)**^ for this review was this: “What is the hepatic elasticity in kPa, obtained by ultrasound elastography, that can predict the risk of a diagnosis of HCC on MRI, CT, or biopsy?”. Studies were included if they compared results in kPa for detecting HCC through ultrasound elastography (p-SWE or 2D-SWE), using MRI, CT, or biopsy as the reference standard.

The inclusion criteria for this study were as follows: having included patients diagnosed with HCC, including parenchymal elasticity measurements obtained via p-SWE or 2D-SWE with results reported in kPa or meters per second, and having included patients who had undergone biopsy, CT, or MRI for diagnostic confirmation. Systematic reviews were excluded, as were studies utilizing transient elastography or FibroScan.

There were no restrictions regarding the origin or publication status of the studies. Articles published in English, Portuguese, or Spanish were included. In cases of incomplete information, the corresponding authors were contacted via email for clarification.

Two authors independently assessed the eligibility of potential studies, with a third author resolving any disagreements. The final selection of full-text articles was thoroughly reviewed to confirm study eligibility. Data extraction was conducted by using a standardized form, which included information on study design, authorship, year of publication, country of origin, sample size, and diagnosis of HCC through ultrasound elastography, specifically p-SWE or 2D-SWE, with one of the following comparator methods serving as the gold standard: MRI, CT, or biopsy.

Studies that included a control group were assessed with the Quality Assessment of Diagnostic Accuracy Studies (QUADAS-2) tool^**(^21^)**^. To evaluate biases and precision in all eligible studies, the Research Triangle Institute (RTI) Item Bank^**(^22^)**^ was utilized.

This systematic review followed several key steps^**(^19^,^20^)**^: formulating the research question; selecting the databases and defining the search period; outlining the search strategies; identifying relevant descriptors; conducting a comprehensive, systematic database search; establishing inclusion criteria for original articles; collecting data; selecting relevant evidence; critically evaluating the eligibility of original articles; excluding those that did not meet the inclusion criteria; assessing the quality of eligible studies; synthesizing the findings; and discussing the limitations of and evidence available in each of the studies selected.

## STUDIES SELECTED

The systematic review initially identified 1,131 papers. After 171 duplicates were excluded, 960 articles remained for further analysis based on their titles and abstracts. A total of 923 studies were excluded, for the following reasons: not involving the use of p-SWE or 2D-SWE (n = 788); being an animal study (n = 33); focusing on an unrelated topic (n = 25); being a review article (n = 19); being a letter to the editor (n = 19); being a comment on another study (n = 15); being an *in vitro* study (n = 9); not including patients diagnosed with HCC (n = 7); being a case report (n = 5); and being an editorial (n = 3).

**Figure 3:**
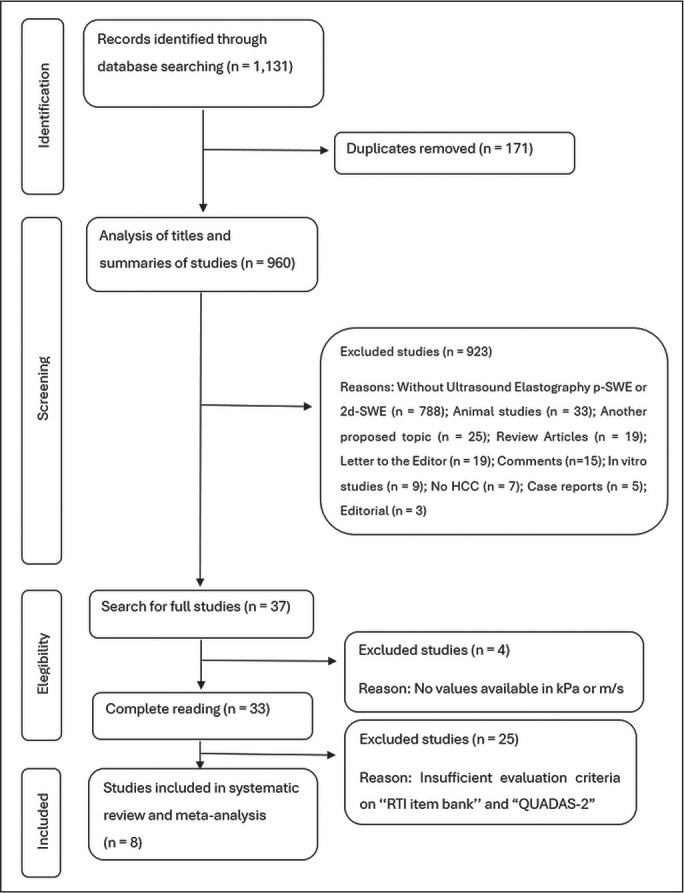
Flow chart of the article selection process.

Of the 37 studies remaining in the analysis, the full texts were thoroughly reviewed, and four articles were excluded because hepatic elasticity values were not reported in kPa or meters per second (Figure 3) and their corresponding authors did not respond to requests for additional information. Consequently, 33 studies met the quality assessment criteria and were carefully analyzed with the RTI Item Bank and QUADAS-2 tools. Of those, eight studies were deemed suitable for inclusion in this systematic review. The limitations and QUADAS-2 scores of the excluded studies are discussed below.

### Characteristics of the studies included

Table 2 shows the characteristics of the eight studies included in the systematic review. Those included a collective total of 501 patients, with a mean age of 57.8 ± 15.8 years. All of the studies included patients diagnosed with HCC by p-SWE or 2D-SWE ultrasound elastography and confirmed by MRI, CT, or biopsy. Of the 501 patients, 76.4% were male. Liver elasticity measurements, expressed in kPa or meters per second, were derived from the hepatic parenchyma rather than the tumor, ensuring a more accurate representation of the overall pathological condition of the liver. Results initially presented in meters per second were standardized to kPa by using the formula *E* = 3 × *V^^2^^*, as described by Graff^**(^23^)**^, a validated method for converting shear wave velocity (*V*) in meters per second into Young’s modulus (*E*) in kPa.

**Table 2:** General characteristics of the studies included.

Reference	Study design	Population with confirmed HCC	Inclusion criteria	Index test	Elasticity (kPa) Mean ± SD
Chou et al.^(^24^)^	Prospective	N = 77; 82% male; mean age, 61.22 ± 13.85 years	Scheduled for elective hepatectomy for HCC, aged > 18 years, with preoperative diagnosis based on imaging or biopsy	Yes	19.06 ± 1.08
Gallotti et al.^(^25^)^	Multicenter prospective	N = 6; gender and age not available	Solid focal liver lesions larger than 1.5 cm and located < 5.5 cm from the liver surface, diagnosed by at least two imaging methods	Yes	14.13 ± 2.17
Grgurevic et al.^(^29^)^	Prospective	N = 57; gender and age not available	Focal liver lesions detected on ultrasound, located in the right liver lobe, and < 7 cm deep, with no ongoing inflammation or liver congestion	Yes	29.57 ± 11.67
Guo et al.^(^30^)^	Prospective	N = 69; 78.3% male; mean age, 56.07 years (interquartile range, 10.51 years)	Solid focal liver lesions diagnosed on conventional ultrasound with a lesion depth < 8 cm and adequate SWE image quality	Yes	13.74 ± 0.63
Hasab-Allah et al.^(^26^)^	Case series confirmed by CT, MRI, or both	N = 143; 75.5% male; mean age, 59.02 ± 6.48 years (range, 40-77 years)	HCC or liver metastases confirmed by imaging or histopathology	Yes	17.05 ± 8.53
Heide et al.^(^27^)^	Prospective	N = 5; sex and age data not available	Indeterminate focal liver lesions on B-mode ultrasound, scheduled for contrast-enhanced ultrasound or biopsy	Yes	25.06 ± 2.94
Kang et al.^(^28^)^	Prospective	N = 38; 58% male; mean age, 62.3 ± 9.75 years	HCC based on the guidelines of the Korea Liver Cancer Study Group, with lesions identified on imaging	Yes	18.15 ± 1.47
Xie et al.^(^31^)^	Prospective	N = 106; 79.2% male; median age, 55 years (interquartile range, 29-76 years)	Diagnosis of HCC confirmed by at least two imaging methods, scheduled for hepatic resection	Yes	15.52 ± 5.86

All eight of the studies evaluated compared ultrasound elastography results with those obtained by MRI, CT, or liver biopsy. In the evaluation of liver stiffness, five of those studies utilized p-SWE^**(^24^-^28^)**^, whereas three employed 2D-SWE^**(^29^-^31^)**^. None of the studies applied vibration-controlled transient elastography as part of the assessment. The maximum interval between the elastography examination and examination with one of the gold-standard methods was 20 days. All of the studies reported differences between elastography and the reference standard in terms of the detection of liver stiffness^**(^24^,^28^-^31^)**^, as well as in terms of identifying benign and malignant lesions in the parenchyma^**(^25^-^27^)**^. Four studies also assessed the elasticity of a nodule or lesion, in addition to hepatic parenchymal elasticity^**(^25^-^27^,^29^)**^. All p-SWE and 2D-SWE studies were interpreted by experienced radiologists.

### Findings

A meta-analysis was conducted to estimate the mean liver elasticity in patients with HCC. The meta-analysis was performed with the Stata SE statistical software package, version 14.1 (StataCorp LP, College Station, TX, USA). All eight of the studies analyzed reported means with standard deviations or medians with interquartile ranges for that parameter. To ensure comparability among the studies, in cases in which the studies reported medians with ranges or interquartile ranges, the appropriate formulas were applied to convert those measures to means with standard deviations^**(^32^)**^.

**Figure 4:**
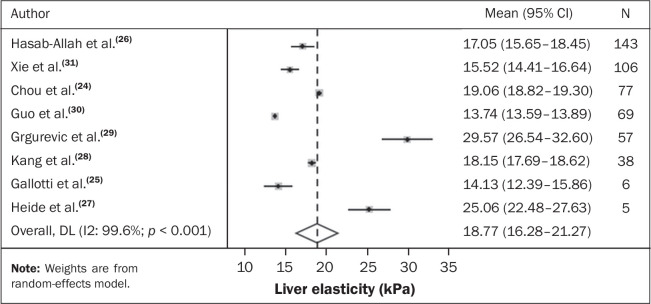
Forest plots of the sensitivity and specificity of 2D-SWE and p-SWE.

Heterogeneity was assessed by using Cochran’s Q test and the Higgins I^^2^^ statistic. To check for the presence of publication bias, a funnel plot was constructed. Given the significant heterogeneity across the studies, the analysis was conducted using a random-effects model. Forest plots (Figure 4) illustrate the sensitivity and specificity of the 2D-SWE and p-SWE techniques. For each study, the results are presented together with 95% confidence intervals (95% CIs), as well as the combined estimate of the mean liver elasticity^**(^32^)**^. In that scenario, among the 501 patients diagnosed in the study, the average elasticity of the liver parenchyma was estimated at 18.77 kPa (95% CI: 16.28-21.27 kPa). This average stiffness value was found to be associated with the risk of developing HCC, potentially reflecting changes in liver elasticity distal to the tumor.

**Figure 5:**
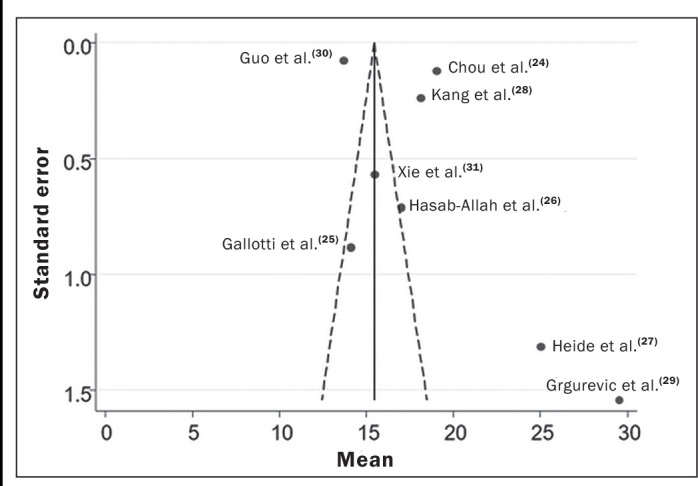
Funnel plots of the standard error of the study data.

The summary estimates potential publication bias and other biases that could affect the meta-analysis results, obtained from the analysis of the 2D-SWE or p-SWE findings. A funnel plot (Figure 5) demonstrates the standard error of the included studies and reflects the observed heterogeneity. Each point of the funnel plot represents an individual study, with the horizontal axis showing the mean elasticity reported and the vertical axis representing the standard error. Asymmetry in the distribution of points suggests significant heterogeneity among the studies, highlighting the importance of this tool in ensuring the validity and reliability of our findings. In our assessment of the robustness of our data, excluding studies from the overall analysis of the risk of bias did not decrease the heterogeneity.

### Methodological quality assessment

The QUADAS-2 tool was applied to assess the methodological quality of all eight of the studies included. The results were classified as having a high risk of bias in two (25%) of the studies regarding applicability issues, specifically in patient selection^**(^31^)**^ and choice of index test^**(^29^)**^, and as having a moderate risk of bias and applicability concerns in six (75%). None of the studies presented a low risk in all four domains. A comprehensive overview of the individual assessments is provided in Table 3.

**Table 3:** QUADAS-2 risk of bias.

Reference	Risk of bias				Applicability concerns		
	Patient selection	Index test	Reference standard	Flow and timing	Patient selection Index test		Reference standard
Hasab-Allah et al.^(^26^)^	?	+	+	?	+	+	+
Grgurevic et al.^(^29^)^	?	?	+	+	+	-	+
Gallotti et al.^(^25^)^	+	?	+	+	+	?	+
Guo et al.^(^30^)^	+	?	+	+	?	+	+
Xie et al.^(^31^)^	?	+	+	+	-	+	+
Kang et al.^(^28^)^	+	+	?	+	+	+	?
Heide et al.^(^27^)^	+	+	?	+	+	?	+
Chou et al.^(^24^)^	?	+	+	+	+	+	?

?: unclear risk; +: low risk; -: high risk.

## DISCUSSION

Ultrasound elastography has been identified in this study as a highly effective technique for evaluating liver stiffness in patients with HCC, as supported by findings in the medical literature^**(^33^,^34^)**^. The p-SWE and 2D-SWE techniques both demonstrated strong accuracy in detecting malignant liver lesions, regardless of lesion depth or patient comorbidities, with minimal variation between the two methods. Notably, 2D-SWE exhibited a slight advantage in detecting deeper lesions, as reported in prior studies^**(^35^)**^, and demonstrated a high (up to 96%) rate of accuracy for distinguishing between malignant and benign focal liver lesions^**(^36^)**^.

Among the 501 patients collectively evaluated in our study, liver stiffness measurements showed adequate sensitivity and specificity for HCC detection, with an average stiffness value of 18.77 kPa, consistent with that reported in previous studies. However, there are no comparable results for hepatic parenchymal elasticity measured by 2D-SWE or p-SWE in HCC patients, unlike transient elastography, which is well-established for outcome prediction in such cases. Although the differences between p-SWE and 2D-SWE were not statistically significant, 2D-SWE provided more robust data for patient and lesion analysis, as also noted by Nacheva-Georgieva et al.^**(^36^)**^.

These findings reflect differences in operational principles among 2D-SWE, p-SWE, and transient elastography (FibroScan). The 2D-SWE and p-SWE techniques both utilize ultrasound to generate shear waves, enabling real-time imaging and localized liver stiffness measurements. This facilitates precise evaluation of specific hepatic regions and enhances the detection of focal lesions, including HCC^**(^36^)**^. Conversely, FibroScan generates shear waves mechanically, measuring overall liver stiffness without real-time imaging, which limits its ability to identify localized abnormalities like tumors^**(^35^)**^.

Previous studies, including those conducted by Grgurevic et al.^**(^29^)**^, Wang et al.^**(^37^)**^, and Silva et al.^**(^38^)**^, demonstrated the effectiveness of elastography in differentiating between benign and malignant liver lesions, which aligns with our findings. Liver stiffness was significantly higher in patients with HCC than in those with benign lesions, as described by Hasab-Allah et al.^**(^26^)**^ and Heide et al.^**(^27^)**^. The ability of elastography to assess liver parenchymal elasticity provides critical information for early HCC diagnosis and lesion differentiation^**(^35^)**^.

Despite the findings of Jiang et al.^**(^39^)**^, who used FibroScan to assess liver stiffness in patients with chronic hepatitis B, our results suggest that p-SWE and 2D-SWE are better suited for evaluating patients already diagnosed with HCC. Jiang et al. reported a median liver stiffness of 7.7 kPa^**(^39^)**^, whereas we identified an average stiffness of 18.77 kPa in HCC patients. The broader and more precise assessment capabilities of p-SWE and 2D-SWE highlight their clinical utility^**(^39^)**^.

Elastography also offers advantages in terms of accessibility and cost in comparison with invasive diagnostic methods like liver biopsy^**(^37^,^40^)**^. As a noninvasive technique, elastography allows efficient screening and monitoring of at-risk populations, making it particularly valuable in resource-limited settings^**(^38^)**^. Its broader implementation in clinical practice could reduce the economic burden of HCC diagnosis^**(^37^,^38^)**^.

Our study has limitations. Notably, there is limited research directly comparing 2D-SWE and p-SWE for measuring hepatic parenchymal elasticity distal to tumors^**(^35^,^40^)**^. In addition, the number of studies included in the meta-analysis was relatively small, and most p-SWE studies were conducted before 2022. Variability in 2D-SWE measurements between systems continues to be a significant limitation, with discrepancies in shear wave speed estimates ranging from 6% to 12% and up to 17.7% at greater depths^**(^35^)**^. Measurement accuracy decreases at greater depths, with less variability having been observed at specific depths, such as 4 cm for convex probes and 3-4 cm for linear probes^**(^35^,^40^)**^.

This systematic review emphasizes the importance of future research to improve elastography as a diagnostic tool, including its integration with advanced techniques for enhanced diagnostic accuracy and treatment personalization^**(^33^,^34^)**^. Multicenter studies with more diverse populations and comorbidities, such as cirrhosis, are also necessary to validate liver stiffness cutoff values^**(^35^,^36^)**^. On the basis of the findings presented here, p-SWE and 2D-SWE elastography appear to be valuable, accessible, and effective tools for early HCC diagnosis, with the potential to improve disease management through earlier diagnoses and interventions^**(^38^-^40^)**^.

## CONCLUSION

This systematic review and meta-analysis demonstrated that ultrasound elastography, using the p-SWE or 2D-SWE technique, may be an effective tool for assessing the risk of HCC in patients with hepatic fibrosis, with a mean liver parenchymal elasticity of 18.77 kPa, as corroborated by previous studies. Elastography has proven capable of differentiating between benign and malignant liver lesions, offering advantages such as accessibility and low cost, making it a complement to MRI, CT, and liver biopsy. Additionally, its application enables regular monitoring of at-risk patients, facilitating early diagnosis and more effective interventions. However, the heterogeneity of the studies included and the limited number of articles in this systematic review and meta-analysis represent limitations, highlighting the need for future prospective studies with larger patient cohorts to validate and optimize the use of elastography in clinical practice. In conclusion, p-SWE and 2D-SWE show significant potential to enhance early HCC diagnosis and to have a positive impact on public health.
